# Hope and Optimism as an Opportunity to Improve the “Positive Mental Health” Demand

**DOI:** 10.3389/fpsyg.2022.827320

**Published:** 2022-02-24

**Authors:** Carlos Laranjeira, Ana Querido

**Affiliations:** ^1^School of Health Sciences, Polytechnic of Leiria, Leiria, Portugal; ^2^Center for Innovative Care and Health Technology (ciTechCare), Polytechnic of Leiria, Leiria, Portugal; ^3^Research in Education and Community Intervention (RECI I&D), Piaget Institute, Viseu, Portugal; ^4^Center for Health Technology and Services Research (CINTESIS), NursID, University of Porto, Porto, Portugal

**Keywords:** hope, positive mental health, mental health literacy, nursing, optimism

## Introduction

As the world confronts the COVID-19 pandemic and its consequences, including an associated mental health crisis, finding meaning and building positive processes and capacities will help strengthen future mental health (Waters et al., 2021). Existential perspectives use this basic insight to advocate that suffering is an inherent part of life that must be confronted, rather than avoided or amended (Israelashvili, 2021). Positive psychology studies adaptation to adversity and aims to identify factors that favour good psychological adjustment, as well as physical and mental health (Reppold et al., 2015; Phan et al., 2020). Notwithstanding, the mental-ill health approach is still predominant, with authors suggesting the need to improve “mental health literacy” and promote access to mental health services (Mansfield et al., 2020).

Recently, there has been increased interest in assessing the attributes, structure and individual variability of wellbeing and identifying its psychological promotors. This research has begun to clarify the determinants of positive mental health (Gallagher and Lopez, 2009; Das et al., 2020). Positive mental health encompasses the personal resources to face life's challenges, foster satisfactory relationships with others, and achieve psychological wellbeing, including feelings of satisfaction with life, vitality and energy, and physical wellbeing (Teixeira et al., 2019). Applied to Meleis (2010) theory, positive mental health can facilitate a healthy changeover within a transitional process, since personal, community, and social conditions can foster or restrict healthy transitions and the outcome of transitions.

There is a need for mental health literacy programs that are focused on hope, and provide accurate information about disorders and recovery. Positive expectations for the future, commonly conceptualised as hope and optimism in the literature, can act as potential mechanisms toward achieving positive mental health (Gallagher and Lopez, 2009, 2018). The conceptualizations of dispositional hope (Snyder, 2002) and dispositional optimism (Scheier and Carver, 1985; Carver and Scheier, 2014) share several elements: (a) personality traits, (b) cognitive constructs, (c) reference to general expectancies, (d) relation to significant personal goals, (e) future orientation, and (f) acting as determinants of behaviour (Krafft et al., 2021). Hope and optimism, although often used interchangeably in clinical discourse, are in fact distinct constructs, corresponding to distinct mechanisms by which expectations shape human behaviour and produce positive outcomes (Gallagher and Lopez, 2009; Schiavon et al., 2017).

This opinion paper aims to examine some differences between optimism and hope, and integrate these constructs in the context of positive mental health. We also intend to point out some interventions that promote hope and optimism, where mental and psychiatric health nursing play an important role.

## Benefits of Optimism And Hope For Positive Mental Health

Whether optimism and hope can affect physical and mental health has been discussed among academics around the world (Milona, 2020). There is empirical evidence supporting the notion that both attitudes contribute to positive outcomes (Schiavon et al., 2017; Pleeging et al., 2021). In this context, optimism is defined as a cognitive variable reflecting one's favourable view about their future (Carver and Scheier, 2019). Optimists generally have more positive than negative expectations and tend to report less distress in their daily lives, even in the face of challenges (Carver et al., 2010). What is expected to happen in the future can affect how people experience situations in their daily lives, their health, and how they deal with emotions and stress.

Optimists are more focused on generalised expectations rather than how or why the goal is achieved (Carver and Scheier, 2002). Studies has found that optimism is related to fewer symptoms of depression, higher levels of wellbeing, lower attrition rates, and stronger perceptions of social support (Forgeard and Seligman, 2012; Schug et al., 2021). The positive repercussions of optimism may be related to the greater probability of adopting health-promoting behaviours and coping strategies that enable better psychic adjustment (Carver and Scheier, 2014, 2019). Recent evidence reveals that optimism is modifiable and associated with better cardiovascular health (Boehm et al., 2020) and increased likelihood of healthy aging (James et al., 2019; Lee et al., 2019). In contrast, pessimists have a less favourable perception of the world and are more likely to adopt risky behaviours, such as the use and abuse of alcohol and other drugs (Carver and Scheier, 2019), and display more harmful reactions and adaptations to adversities, compared to optimists (Forgeard and Seligman, 2012; Carver and Scheier, 2019).

Snyder (1991, as cited in Schiavon et al., 2017, p. 2) defined “hope as a state of positive motivation based on three components: objectives (goals to be achieved), pathways (planning to achieve these goals), and agency (motivation directed toward these objectives).” Hope theory emphasises the presence of personal agency related to goals and the recognition of strategies to achieve those goals (Snyder, 2002). Therefore, this theory suggests that a hopeful person would endorse statements such as “I will achieve my goal,” but also “I have a plan […] to achieve this goal” and “I am motivated and confident in my ability to use this plan to achieve this goal” (Gallagher and Lopez, 2009, p. 548). According to recent research, an individual's level of hope is often determined by innate personality characteristics and influenced by psychosocial conditions (success in attaining goals and facing stressors, social support, goal-concordant care), physiological factors (including stress hormones, immune mediators, and neurotransmitters) and environmental factors (Corn et al., 2020).

Snyder (2002) hope theory is not the only perspective that distinguishes hope from optimism: Herth's model of hope assumes that hope is a cognitive and motivational attribute needed to initiate and support action towards goal achievement (Arnau et al., 2010).

Currently, mental health literacy programs aim to understand how to reach and maintain positive mental health, recognise mental disorders and their beliefs about treatments, reduce stigma towards mental disorders, and enhance the help-seeking ability, namely when and where to seek help, but also how to best manage and improve one's own mental health (Kutcher et al., 2016). As a catalyst for positive change, hope promotes overall mental health and may help heal specific conditions, including severe mental illness, suicidal ideation, depression, anxiety and trauma-related disorders (Huen et al., 2015; Gallagher et al., 2020; Tomasulo, 2020; Sari et al., 2021). Research also demonstrates that hope promotes wellbeing more than optimism or self-efficacy (individual's belief in their own ability perform task and attain goal) (Krafft et al., 2021). In addition, research shows that hope has strong associations with several psychosocial process and outcomes, including positive affect, emotional adjustment and illness-related coping, greater life satisfaction, enhanced perceptions that life is meaningful, a higher sense of purpose in life, quality of life, and social support (Corn et al., 2020; Long et al., 2020).

A large longitudinal study among older adults exploring the potential public health implications of hope for subsequent health and wellbeing outcomes revealed that “a greater sense of hope was associated with: better physical health and health behavior outcomes (e.g., reduced risk of all-cause mortality, fewer chronic conditions, and fewer sleep problems), higher psychological wellbeing (e.g., increased positive affect, life satisfaction, and purpose in life), lower psychological distress, and better social wellbeing” (Long et al., 2020, p. 1).

Long-term, mental health promotion in vulnerable populations is deeply intertwined with hope-based interventions. At a time when predictions regarding mental health are particularly grim, those involved in promoting mental health, need to pay close attention to the relation between evidence, hope and intervention.

## Strategies That Promote Hope and Optimism in Psychiatric-Mental Health Nursing

Psychiatric-mental health nursing is grounded in interpersonal engagement and includes a broad range of helping activities, from teaching to counselling (Hartley et al., 2020). Within this interpersonal context, it is well known that expectations about the future directly influence the subject's wellbeing. Given the protective effect of optimism and hope in people's lives, especially with regard to better physical and mental health, two questions must be considered: is it possible to develop or enhance levels of optimism and hope? How they can be mobilized as an intra/interpersonal healing resource?

The techniques grounded in cognitive-behavioural therapy can be an effective strategy to develop more positive beliefs about the future. Using this approach, nurses can help their patients understand the schemes that coordinate their thoughts, behaviours and feelings (Carver and Scheier, 2014). Optimism can be activated by training and cognitive restructuring regarding the subject's way of thinking and acting (Carver et al., 2010; Carver and Scheier, 2014).

The evidence suggests the possibility of applying optimism and hope through different strategies and intervention programs (Malouff and Schutte, 2016), especially psychoeducation and cognitive restructuring strategies aimed at extracting positive aspects of everyday situations. Testing such interventions is a first step toward producing and spreading effective programs that underscore the positive effects of optimism (Carver and Scheier, 2014).

Mental health, hope and optimism are intimately related, and can be reinforced with simple daily actions that boost mental strength, even in the midst of uncertainty such as currently faced with the COVID-19 pandemic. Thus, we recommend some evidence-based practices to promote hope/optimism and support better mental health literacy (Table 1).

**Table 1:** Strategies to cultivate a hope/optimism mind-set (based on Newport Academy, 2020).

Emphasis on strengths	Identifying and exploring individual strengths fosters a sense of hope and resilience. Bonding with others is one of our strengths, thus reaching out to friends and loved ones can create hope and positive emotions (Pleeging et al., 2021)
Reframe negative thoughts	When a person feels anxious or desperate, focusing on what is scary and seeing it in a positive way can bring an immediate sense of pleasure and pride in themselves (Das et al., 2020). For example, if we think “I'm never going to stop feeling anxious about everything that's going on,” we can shift to “It's normal to be anxious, and there are things I can do to make it better”
Practice hopeful thinking	A focus on hopeful thinking as an intervention enabling individuals to reengage in pleasant activities and improve self-talk. Hopeful thinkers take intentional action to achieve a desired outcome. Practice of hopeful thinking involves the perceived capacity to envision workable routes together with the energy towards goals attainment (Snyder, 2002). In depressed individuals, practising hopeful thinking decreases symptoms of sadness and depression and improves meaning in life, happiness and wellbeing (Gallagher and Lopez, 2018)
Increasing self-esteem and self-awareness	Mindfulness-based interventions—including activities like sensory awareness, guided meditation, breath control—foster happiness and self-awareness (Goldberg et al., 2018). When a person feels optimistic and hopeful, they often view themselves as benefiting from another person's generosity, leading them to feel valued. This increases self-esteem, which in turn leads to higher levels of psychological wellbeing (Allen, 2018)
Hang out with hopeful and optimistic people	Surrounding ourselves with hopeful and positive people can, by “emotional contagion,” lead us feel that way ourselves. Evidence reveals that both positive and negative emotions are “contagious,” so we need to choose our social environment and interactions (Herrando and Constantinides, 2021)
Practice gratitude	Simple gratitude practices—like journaling, self-compliments, or sending thank you notes—can bring sanctity and authentic happiness (Bohlmeijer et al., 2021) and be more effective than self-control, patience, or forgivingness in generating hope for the future
Reinforcing positive affect	Induced positive affect (by several different means such as viewing a comedy film, receiving a gift) facilitates flourishing and predicts subjective wellbeing. Hope is related with positive affect and inversely with negative affect (Gallagher and Lopez, 2018). Positive emotions are particularly important to mental health in the context of high stress (Israelashvili, 2021)
Training resilience and finding a sense of purpose	Resilience refers to the ability to recover quickly from adverse events and experiences. Resilient people tend to maintain a more positive outlook and cope with stress more effectively (Vos et al., 2021). Facing crises can be strengthened by finding a sense of purpose in life. This might implicate involvement in the community, cultivating spirituality or participating in meaningful activities (Manning et al., 2019)

## Conclusion

In sum, optimism and hope are important adaptive phenomena that foster wellbeing, quality of life, and psychological adjustment in the general population and in specific groups, such as people living with mental health conditions. Optimistic and hopeful individuals adapt better to adversity, have lower chances of developing mental disorders, and exhibit behaviours that are healthier and related to greater satisfaction with life. Given these benefits, understanding how hope and optimism arise and flourish is of great interest, and will help develop promotors of mental health. More evidence is needed to develop hope-based interventions and establish their true efficacy. Ideally, these studies would involve randomized control trials (RCTs) with appropriate sample sizes that compare optimism and hope-based interventions to already validated gold standard treatments.

## Author Contributions

All authors listed have made a substantial, direct, and intellectual contribution to the work and approved it for publication.

## Funding

This work is funded by national funds through FCT—Fundação para a Ciência e a Tecnologia, I.P. (UIDB/05704/2020 and UIDP/05704/2020) and under the Scientific Employment Stimulus—Institutional Call (CEECINST/00051/2018).

## Conflict of Interest

The authors declare that the research was conducted in the absence of any commercial or financial relationships that could be construed as a potential conflict of interest. The handling editor declared a shared affiliation with one of the authors AQ at time of review.

## Publisher's Note

All claims expressed in this article are solely those of the authors and do not necessarily represent those of their affiliated organizations, or those of the publisher, the editors and the reviewers. Any product that may be evaluated in this article, or claim that may be made by its manufacturer, is not guaranteed or endorsed by the publisher.
